# Therapeutic drug level of tacrolimus causing intracranial hemorrhage in a patient with renal transplant

**DOI:** 10.1002/ccr3.5788

**Published:** 2022-04-26

**Authors:** Sangam Shah, Rajeev Ojha, Rajan Chamlagain, Santosh Chhetri, Pravin Prasad, Bikash Baral, Bindu Gyawali, Ashish Shrestha, Jayant Kumar Yadav

**Affiliations:** ^1^ Maharajgunj Medical Campus Institute of Medicine Tribhuvan University Maharajgunj Nepal; ^2^ Department of Internal Medicine Institute of Medicine Tribhuvan University Maharajgunj Nepal; ^3^ Department of Neurology Institute of Medicine Tribhuvan University Maharajgunj Nepal; ^4^ Department of Nephrology Institute of Medicine Tribhuvan University Maharajgunj Nepal; ^5^ Department of Clinical Pharmacology Institute of Medicine Tribhuvan University Maharajgunj Nepal

**Keywords:** intracranial hemorrhage, renal transplant, tacrolimus

## Abstract

Tacrolimus is used in solid organ transplant patients to prevent rejection, and no case of intracerebral hemorrhage (ICH) has been reported till date. We report a case of 31‐year‐old man with diabetes and hypertension for ten years who had a renal transplant four years back; diagnosed with tacrolimus‐induced ICH.

## Graphical abstract

Tacrolimus causing intracranial hemorrhage has not been reported. It is to be considered as one of the differential diagnosis of intracranial hemorrhage after ruling out the more common causes.

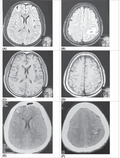

## 1 INTRODUCTION

Tacrolimus, a calcineurin inhibitor, is used after solid organ transplantation to prevent graft rejection.^1^ Tacrolimus can cause several side effects like infection, hypertension, hypomagnesemia, hyperkalemia, blurring of vision, itching, hyperglycemia, and is nephrotoxic.^2^ It also causes neuropsychiatric problems such as insomnia, posterior reversible encephalopathy syndrome, confusion, weakness, depression, cramps, neuropathy, seizure, tremors, and catatonia. However, Tacrolimus causing intracerebral hemorrhage (ICH) has not been reported till date. We report a case of a 31‐year‐old man with tacrolimus‐induced ICH who had undergone renal transplant four years ago.

## 2 CASE PRESENTATION

A 31‐year‐old man was admitted to our hospital with chief complaints of fever, vomiting, and altered sensorium for four days. Fever was not associated with chills and rigor; vomitus was non‐bilious and projectile. He had irrelevant talk, and was not able to recognize his own family members. He had no history of trauma. He had undergone renal transplant four years back for Chronic Kidney Disease (CKD) stage V and was on medications: Tacrolimus (1 mg and 0.5 mg in morning and evening, respectively), Mycophenolate mofetil (500 mg three times a day), and Prednisolone (5mg once a day). He had diabetes and hypertension for 10 years for which he was taking gliclazide (40mg once a day), metformin (500 mg once a day), and amlodipine (5 mg OD). He smoked 3–4 cigarettes per day and consumed alcohol daily for the last 13 years. He had not taken any over the counter medication drug.

During the initial visit, he was not oriented to time, place, and person, and Glasgow Coma Scale was 9/15 (E_2_V_2_M_5_). On general physical examination, he had no cyanosis, edema, icterus, pallor or clubbing. His body temperature was 103^◦^F, blood pressure was 130/70 mm Hg, pulse rate was 77 beats per minute, and respiratory rate was 18 breaths per minute. Power was 1/5 in the shoulder abduction and adduction, elbow flexion and extension, and wrist flexion and extension of right hand. Furthermore, the hip flexion and extension, knee flexion and extension, and ankle flexion and dorsiflexion of the right leg were 2/5. Neck rigidity, and Kernig sign were present but Brudzinski sign was absent.

Laboratory examination revealed hemoglobin 16 gm % and hematocrit of 47.3%. Prothrombin time (PT) and International Normalized Ratio (INR) was 13 s and 1.14, respectively, and bleeding time (BT), clotting time (CT) and thyroid hormone level were also in normal range. His total leukocyte count was 17,710 cells/mm^3^, neutrophils 83%, lymphocytes 6%, monocytes 11%, and platelet count 1,26,000 cells/mm^3^. The patient was tested negative for COVID‐19. His random blood sugar level was 165 mg/dl and both total protein (5.9 g/dl) and serum albumin level (2.5 g/dl) were decreased. The blood was drawn after three days of last dose of Tacrolimus and its level in the blood was 3.5 µg/L. Gram‐positive *Staphylococcus aureus* was isolated in blood culture. Cerebrospinal Fluid (CSF) analysis showed a total leucocyte count of 35 cells/mm^3^ with predominant lymphocytes (100%), normal glucose (5.1 mmol/L), and protein level (83 g/L). Procalcitonin level was 0.17 ng/ml. Brucella antibody titer was <180 (titer >1:80 is significant) and C ‐ reactive protein was positive using Latex Particle Agglutination Test. Scrub typhus antibody, leptospira antibody, and cryptococcal antigens were not detected by immunochromatography. Herpes simplex virus (HSV) was not detected in CSF Polymerase Chain Reaction.

**FIGURE 1:**
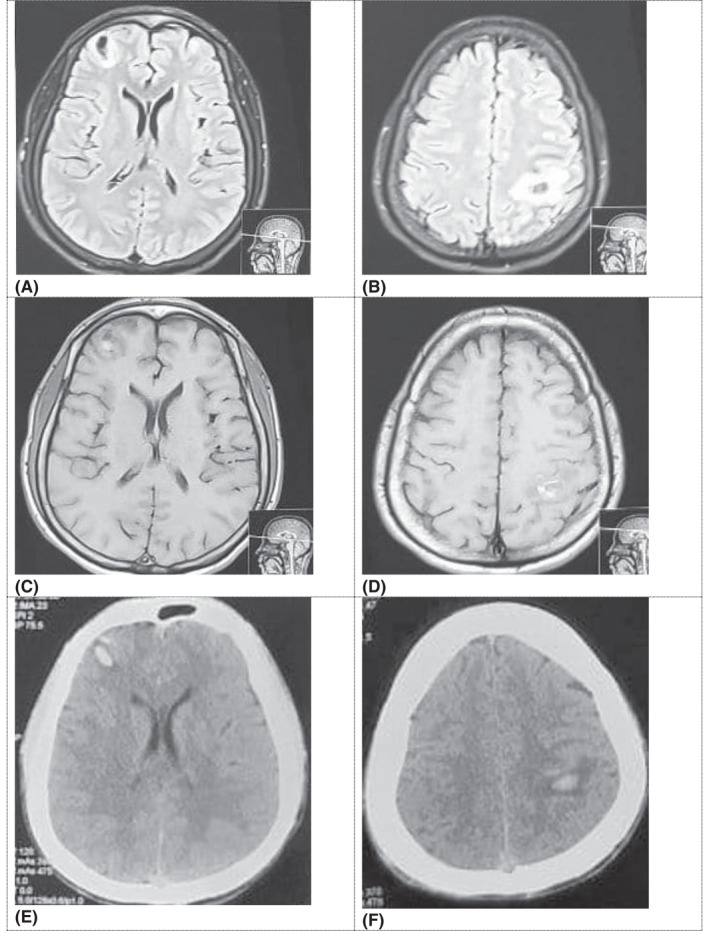
(A, B) FLAIR Axial images shows central hypointense with surrounding hyperintense lesion in right frontal and left parietofrontal region; (C, D): T1‐weighted axial shows central hyperintense with surrounding hypointense lesion in right frontal and left parietofrontal region: (E, F) CT head plain shows central hyperdense with surrounding hyperdense lesion in right frontal and left parietofrontal region

High‐Resolution Computed Tomography of the thorax and abdomen showed ground‐glass opacities in bilateral lower lung lobes, minimal bilateral pleural effusion with subsegmental atelectasis, mediastinal lymphadenopathy (approximately 13 mm in the right paratracheal region) with the features suggestive of infective pathology. It also revealed hepatomegaly (liver approximately 18 cm craniocaudal). The kidneys were smaller on both sides (right kidney approximately 7.2 × 3 cm and left kidney approximately 6.7 × 3.2 cm). Ultrasonography of the neck showed a normal thyroid scan. Magnetic Resonance Imaging (MRI) of the brain showed multiple focal central hyperintense lesions in T1 and hypointense lesion in T2 and T2 FLAIR, with restricted diffusion noticed in perilesional region in bilateral cerebral hemispheres with the features suggestive of hemorrhage of larger lesions in the right frontal (2 × 1.3 cm) and left parietal lobes (3 × 2.5 cm) with perilesional infarcts (Figure 1). Magnetic Resonance Angiography and Magnetic Resonance Venography brain showed hypoplastic right vertebral artery and left transverse sinus, sigmoid sinus, and jugular vein, respectively, otherwise no significant stenosis.

The patient was diagnosed with meningoencephalitis, bilateral lower lobe pneumonia with ICH. Following this, the patient was managed in the Intensive Care Unit. He was treated empirically with IV acyclovir, vancomycin, ampicillin, ceftriaxone, and doxycycline for possible viral and bacterial infections along with intravenous mannitol for raised intracranial pressure. Insulin was started for the maintenance of blood sugar level. After suspicion of drug induced toxicity, therapeutic drug monitoring using Chemiluminescence Immuno‐Assay (CLIA) was done that revealed 3.5 µg/l level of Tacrolimus (Ref range: 2–30 ng/ml). However, due to clinical suspicion and no other apparent cause for the ICH it was stopped. Patient had 3 episodes of generalized tonic clonic seizures for which levetiracetam and phenytoin were added. After fourteen days of hospital stay, laboratory investigations improved to normal range and Tacrolimus level in the blood was 1.2 µg/L. He was discharged on oral medications (Prednisolone, phenytoin, levofloxacin, aspirin, atorvastatin, and metformin) with normal sensorium and without any focal deficits. On follow‐up after 1 month, the patient was doing well with no new issues.

## 3 DISCUSSION

Few cases of ICH after organ transplantation have been reported in the setting of Posterior Reversible Encephalopathy Syndrome but ICH due to Tacrolimus has not been reported till now.^3^, ^4^ Therefore, establishing tacrolimus as a cause of cerebral hemorrhage was challenging. We excluded all the common possible causes of cerebral hemorrhage to arrive at this diagnosis.

There are many potential causes of cerebral hemorrhage, Hypertension being the most common. Coagulopathies (inherited or acquired), subarachnoid hemorrhage, arteriovenous malformations, neoplasm, and amyloid angiopathy are other possible etiologies commonly found in such patients. Hypertension increases pressure on the small arteries branching from middle cerebral, thalamic, and pontine arteries, but our patient's MRI revealed no features of hyperplasia, degeneration, and necrosis of the small arteries. Likewise, the patient had normal BP maintained throughout after transplantation and also at the time of presentation. Hence, hypertension was ruled out as a possible cause of cerebral hemorrhage. Our patient was not taking any anticoagulants and his normal platelets, BT, CT and PT/INR level ruled out the possibilities of any coagulopathy. There was no feature of subarachnoid hemorrhage or aneurysm in neuroimaging and CSF findings.^5^ HSV encephalitis could also be the cause of hemorrhage.^6^ However, HSV was not detected in the CSF and imaging finding was not consistent with typical HSV lesions.

Arteriovenous malformations, capillary telangiectasias, developmental venous anomalies and cavernous malformations that results from abnormal fragile blood vessels may also cause intracerebral hemorrhage.^7^ However, arteriography and venography study showed normal arterial and venous walls. Amyloid angiopathy commonly occurs in old age and in a patient with high blood pressure.^8^ However, there was no evidence of amyloid deposition on MRI. Neoplastic tissues are hypervascular, and blood vessels are usually fragile that can undergo necrosis, and put the patients at risk of intracerebral bleeding. However, imaging findings ruled out primary neoplasm of the brain as well as metastatic lesions from melanomas, lungs, kidneys, and thyroid.^9^ Drugs causing 6cerebral hemorrhage were the only possible cause left, and upon laboratory investigation, it revealed increased tacrolimus level in blood suggesting a possible association.

Tacrolimus can cause several neurologic complications, out of which, headache is the most frequent adverse event. Tacrolimus causing cerebral bleeding is relatively rare in patients with renal transplant. Tacrolimus‐induced ICH has been reported in patients have therapeutic level of drug concentration as well.^10^ Concomitant use of drugs like diltiazem, co‐trimoxazole, clarithromycin, metoclopramide, cimetidine, cyclosporine, methylprednisolone (corticosteroids, which was also being taken by the patient), omeprazole, etc. can cause further increase in serum tacrolimus level. Our patient was taking diltiazem and prednisone, which inhibited the metabolism of tacrolimus, dramatically increasing the tacrolimus level and causing its toxicity.^11^ Tacrolimus like other calcineurin inhibitors may cause direct injury to the endothelial cells leading to alteration of blood‐brain barrier and release of vasoconstrictors causing vasospasm and hypo‐perfusion. Further, by damaging BBB, it induces dysfunction and increases permeability of BBB causing vasogenic edema. This, in time, may progress to hemorrhage. Also, toll‐like receptor‐4 signaling induces vascular inflammation after the vessels are injured.^12^, ^13^ Cerebral Micro Bleeds (CMBs) could have direct effects on cognitive function and may indicate a risk of future symptomatic intracerebral hemorrhage.^14^, ^15^ Our patient could have asymptomatic cerebral microbleeds in the past and this might have resulted in intracerebral hemorrhage.

Though CLIA is one of the renowned methods for assessing the plasma levels of drugs, the method has its own limitation (non‐specific, cross‐reaction with metabolites) and better methods like LC‐MS/MS is recommended. When elimination of tacrolimus is impaired, its metabolites accumulate in the blood and may result in high levels of drug in the serum.

Causality assessment of tacrolimus‐induced intracerebral hemorrhage was done using Naranjo algorithm and revealed a score of 7, which is classified as probable (Table 1).^16^

**TABLE 1:** Causality assessment of tacrolimus‐induced intracerebral hemorrhage using Naranjo algorithm

Question	Yes	No	Do Not Know	Score
1. Are there previous conclusive reports on this reaction?	+1	0	0	+1
2. Did the adverse event appear after the suspected drug was administered?	+2	−1	0	+2
3. Did the adverse event improve when the drug was discontinued or a specific antagonist was administered?	+1	0	0	+1
4. Did the adverse event reappear when the drug was readministered?	+2	−1	0	0
5. Are there alternative causes that could on their own have caused the reaction?	−1	+2	0	+2
6. Did the reaction reappear when a placebo was given?	−1	+1	0	0
7. Was the drug detected in blood or other fluids in concentrations known to be toxic?	+1	0	0	0
8. Was the reaction more severe when the dose was increased or less severe when the dose was decreased?	+1	0	0	0
9. Did the patient have a similar reaction to the same or similar drugs in any previous exposure?	+1	0	0	0
10. Was the adverse event confirmed by any objective evidence?	+1	0	0	1
Total score				7

Tacrolimus‐induced ICH can be managed by reducing the dose of tacrolimus. But the transplant rejection as a consequence is to be monitored. Proper therapeutic drug monitoring helps to balance therapeutic efficacy and the occurrence of adverse events. Another immunosuppressant, everolimus, can be used to prevent transplant rejection, which has less adverse effects compared to Tacrolimus.^17^ Cases of cerebral hemorrhage by the use of Everolimus have not been reported in the literature till date.

## 4 CONCLUSION

Tacrolimus‐induced intracerebral hemorrhage is a diagnosis of exclusion. It is important to rule out other possible causes before arriving at this diagnosis. Clinicians should bear in mind, the possible interaction between drugs when prescribing for patients with comorbidities. Concomitant use of drugs that increase the level of tacrolimus should be replaced with alternative safe drug. Therapeutic drug monitoring should be done to check the possibility of drug toxicity when suspicions arise.

## CONFLICT OF INTEREST

Authors have no conflicts of interest to disclose.

## AUTHOR CONTRIBUTIONS

RO conceptualized the study, reviewed, edited the manuscript, and was in charge of the case; SS wrote the original, reviewed and edited the manuscript; SS, RO, RC, SC, PP, BB, BG, JKY and AS were in charge of the case, and reviewed the manuscript.

## ETHICAL APPROVAL

There was no ethical issues as written informed consent was obtained from the patient.

## CONSENT

Written informed consent was obtained from the patient for publication of the case report.

## Data Availability

All the required information is in manuscript itself.
